# Positive species interactions structure rhodolith bed communities at a global scale

**DOI:** 10.1111/brv.13148

**Published:** 2024-09-19

**Authors:** Fabio Bulleri, Nadine Schubert, Jason M. Hall‐Spencer, Daniela Basso, Heidi L. Burdett, Ronaldo B. Francini‐Filho, Jacques Grall, Paulo A. Horta, Nicholas A. Kamenos, Sophie Martin, Matteo Nannini, Pedro Neves, Irene Olivé, Viviana Peña, Federica Ragazzola, Cláudia Ribeiro, Eli Rinde, Marina Sissini, Fernando Tuya, João Silva

**Affiliations:** ^1^ Dipartimento di Biologia Università di Pisa Via Derna 1 Pisa 56126 Italy; ^2^ Centre of Marine Sciences (CCMAR/CIMAR LA), Campus de Gambelas Universidade do Algarve Faro 8005‐139 Portugal; ^3^ Shimoda Marine Research Center University of Tsukuba Shizuoka Japan; ^4^ School of Biological and Marine Sciences University of Plymouth Plymouth UK; ^5^ Department of Earth and Environmental Sciences University of Milano–Bicocca, CoNISMa Research Unit of Milano–Bicocca Milan Italy; ^6^ Umeå Marine Sciences Centre Umeå University Norrbyn Sweden; ^7^ Department of Ecology and Environmental Sciences Umeå University Umeå Sweden; ^8^ Laboratório de Biodiversidade e Conservação Marinha, Centro de Biologia Marinha (CEBIMar) Universidade de São Paulo (USP) São Sebastião Brazil; ^9^ UAR 3113 OSU Institut Universitaire Européen de la Mer, Univ Brest Plouzané France; ^10^ Laboratório de Ficologia, Departamento de Botânica, Centro de Ciências Biológicas Universidade Federal de Santa Catarina Florianopolis Brazil; ^11^ UMR 7144 Adaptation et Diversité en Milieu Marin CNRS, Sorbonne Université, Station Biologique de Roscoff Roscoff France; ^12^ Department of Integrative Marine Ecology Stazione Zoologica Anton Dohrn Villa Comunale Naples NA 80121 Italy; ^13^ Observatório Oceânico da Madeira, Agência Regional para o Desenvolvimento da Investigação Tecnologia e Inovação (OOM/ARDITI) Funchal Madeira Portugal; ^14^ BioCost Research Group, Faculty of Sciences University of A Coruña rúa da Fraga 10 A Coruña 15008 Spain; ^15^ Department of Integrative Marine Ecology Genoa Marine Centre, Stazione Zoologica Anton Dohrn 9 Villa del Principe, Piazza del Principe 4 Genoa 16126 Italy; ^16^ NBFC, National Biodiversity Future Center Palermo 90133 Italy; ^17^ IFCN—Instituto das Florestas e Conservação da Natureza, IP‐RAM Funchal Madeira Portugal; ^18^ Norwegian Institute for Water Research Oslo Norway; ^19^ Department of Marine Biology Federal Fluminense University Niteroi Rio de Janeiro Brazil; ^20^ Grupo en Biodiversidad y Conservación (IU‐ECOAQUA) Universidad de Las Palmas de Gran Canaria Telde Spain

**Keywords:** rhodoliths, encrusting coralline algae, foundation species, maerl beds, benthic habitats, facilitation cascades, marine biodiversity

## Abstract

Rhodolith beds are diverse and globally distributed habitats. Nonetheless, the role of rhodoliths in structuring the associated species community through a hierarchy of positive interactions is yet to be recognised. In this review, we provide evidence that rhodoliths can function as foundation species of multi‐level facilitation cascades and, hence, are fundamental for the persistence of hierarchically structured communities within coastal oceans. Rhodoliths generate facilitation cascades by buffering physical stress, reducing consumer pressure and enhancing resource availability. Due to large variations in their shape, size and density, a single rhodolith bed can support multiple taxonomically distant and architecturally distinct habitat‐forming species, such as primary producers, sponges or bivalves, thus encompassing a broad range of functional traits and providing a wealth of secondary microhabitat and food resources. In addition, rhodoliths are often mobile, and thus can redistribute associated species, potentially expanding the distribution of species with short‐distance dispersal abilities. Key knowledge gaps we have identified include: the experimental assessment of the role of rhodoliths as basal facilitators; the length and temporal stability of facilitation cascades; variations in species interactions within cascades across environmental gradients; and the role of rhodolith beds as climate refugia. Addressing these research priorities will allow the development of evidence‐based policy decisions and elevate rhodolith beds within marine conservation strategies.

## I. INTRODUCTION

Advances in our understanding of the mechanisms that underpin patterns of species distribution and abundance show that positive species interactions are a driving force in the organisation of natural communities (Bertness & Callaway, 1994; Stachowicz, 2001; Bruno, Stachowicz & Bertness, 2003; Michalet *et al*., 2006; Brooker *et al*., 2008; Gross, 2008; Bulleri *et al*., 2016). Foundation species, defined as those often making up most of the biomass in an ecosystem and located at or near the base of directional networks of mutualistic or non‐trophic interactions (*sensu* Ellison, 2019), including trees, corals, seagrasses, oysters, mussels, salt‐marsh plants and seaweeds, can sustain highly biodiverse habitats (Altieri, Silliman & Bertness, 2007; Silliman *et al*., 2011). The physical structure of these foundation species enhances the establishment and persistence of other species *via* three mechanisms: (*i*) amelioration of environmental stress; (*ii*) reduction of consumer or competition pressure; and (*iii*) increased resource availability. These mechanisms support the more generic notion of foundation species as habitat‐formers (Gribben *et al*., 2019).

Many marine communities are structured by a hierarchy of positive interactions triggered by the presence of foundation species (Bruno & Bertness, 2001). For example, on intertidal cobble beaches, shading and substratum stabilisation by the cordgrass, *Spartina alterniflora*, promotes the presence of other species, such as mussels, snails and seaweeds (Altieri *et al*., 2007). Likewise, cockles can expand seaweed distribution onto sandy or muddy bottoms by providing hard substrata for attachment (Gribben *et al*., 2009). Species directly sustained by foundation species can, in turn, act as secondary facilitators, generating a facilitation cascade (*sensu* Altieri *et al*., 2007). The presence of the basal or primary facilitator is a prerequisite for the presence of further habitat‐formers (secondary or upper‐level facilitators). This establishes the hierarchical structure of the whole community, ultimately enhancing the availability of microenvironments and/or resources (Thomsen *et al*., 2010, 2018). Facilitation cascades are often size‐structured (*sensu* Thomsen *et al*., 2016), whereby body size decreases progressively when moving from the basal to upper‐level facilitators. For example, mangrove trees, seagrass or salt‐marsh plants are generally larger than the bivalves or macroalgae they support and that function as secondary facilitators for other invertebrates (Edgar & Robertson, 1992; Altieri *et al*., 2007; Bishop *et al*., 2012). However, in marine environments, facilitation cascades in which relatively small‐bodied foundation species, such as mussels, cockles or tubeworms, support larger secondary facilitators, often macroalgae, have been broadly documented (Witman 1987; Bulleri & Airoldi, 2005; Thomsen *et al*., 2016, 2022; Bracken, 2018; Ape *et al*., 2018). Independently from the size‐structure of the cascade, this chain or web of positive interactions often culminates with species that do not form habitat, but may be relevant as key elements for ecosystem functioning.

Facilitation cascades have been documented in a variety of terrestrial and marine systems globally, including coral reefs, temperate and tropical forests, salt marshes, soft‐bottoms, seagrass meadows, mangroves and kelp forests (Crain & Bertness, 2006; Thomsen *et al*., 2010, 2018, 2022; Gribben *et al*., 2019). For instance, on tidal mudflats, aerial mangrove roots provide substrata for oyster recruitment and entangle drifting algae, which, in turn, generate suitable habitat for a variety of gastropods (Bishop *et al*., 2012). Similarly, epiphytes growing on trees, seagrasses or freshwater plants can host diverse assemblages of invertebrates (Angelini & Silliman, 2014; Thomsen *et al*., 2022). So far, cascades of up to five levels of co‐occurring habitat‐formers have been documented (Thomsen *et al*., 2016).

**Fig. 1:**
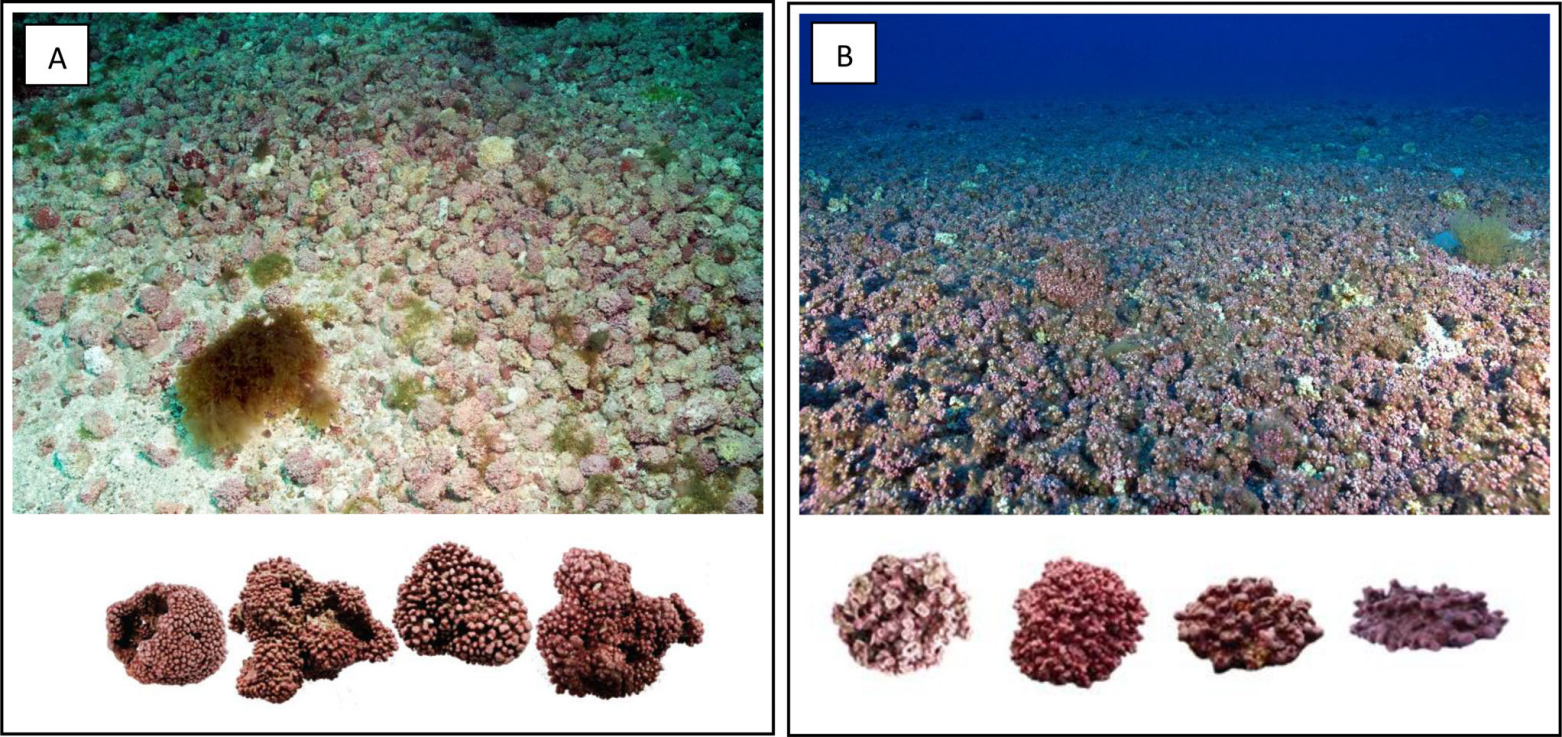
(A) Rhodolith beds from the Fernando de Noronha Archipelago, Brazil, ~ 20 m depth (photograph credit: Ronaldo Francini‐Filho). (B) Rhodolith beds from the Madeira Archipelago, Portugal (photograph credit: Pedro Neves). Bottom images illustrate maerl and rhodolith nodules of different shapes (photograph credits: Eli Rinde and João Silva).

Several aspects of marine facilitation cascades, including underlying mechanisms, spatial configuration (embedded *versus* adjacent), trait‐ and density‐mediated effects, variation across environmental gradients and the relationship between cascade length and temporal stability, have been reviewed recently (Gribben *et al*., 2019). However, out of 100 papers reviewed, none dealt with habitats formed by free‐living red coralline algae (Gribben *et al*., 2019). These so‐called rhodolith or maerl beds are globally distributed and can be highly biodiverse (Fig. 1). This would indicate that the role of rhodoliths as basal habitat‐formers has been overlooked by the ecological facilitation scientific community (but see Otero‐Ferrer *et al*., 2019). Despite rhodolith beds being 2.5–30 times more extensive than habitats formed by widely recognised coastal foundation species (e.g. mangroves, seagrasses and kelps), the number of studies on rhodolith beds is disproportionately small (Rendina *et al*., 2022; Tuya *et al*., 2023), leaving a significant gap in our understanding of their ecological structure, community assembly rules and ecosystem functioning.

Herein, we aim to (*i*) summarise patterns of species and functional diversity of rhodolith beds and their associated communities, (*ii*) identify the mechanisms underpinning rhodolith modification of biotic and abiotic conditions and resource availability, (*iii*) review evidence for the role of rhodoliths as basal species and, hence, as promoters of hierarchical organisation of associated communities, (*iv*) identify their role in sustaining biodiversity and ecosystem functioning, and (*v*) highlight priority research needs and future directions to assess facilitation cascades in rhodolith beds.

## II. RHODOLITH AND MAERL BEDS

The term rhodolith (*rhodo* = red + *lith* = stone) was formally coined by Bosellini & Ginsburg (1971) as “rhodolite”, later corrected to the current spelling to avoid confusion with a garnet. In their original definition, the name referred to unattached nodules formed by calcareous red algae and their branched growths, as part of a continuous spectrum of forms and structures, with size spanning from 2 to 250 mm mean diameter. The term rhodolith parallels the names of other types of unattached nodules composed by different organisms – such as bryolith for unattached bryozoan nodules (James, Foster & O'Sullivan, 2006), corallith for unattached coral nodules (Glynn, 1974) – using nomenclature based on the nodule builder – and includes both nucleated and non‐nucleated nodules of calcareous red algae.

The term “maerl” comes from a Breton word, referring to an area of calcareous land or marine deposits of calcified algae (Grall & Hall‐Spencer, 2003). In northeastern Atlantic countries, the term maerl has been used for centuries to indicate mostly twig‐like, frequently intertwined, unattached coralline algae forming thick accumulations on the shallow seabed. Based on these definitions, the term “rhodolith beds” includes beds made of nucleated and non‐nucleated nodules (i.e. maerl beds) and calcareous *Peyssonnelia* beds (Steller *et al*., 2003; Steller & Foster, 1995; Foster *et al*., 2013). Herein, we will use “rhodoliths” to include both rhodolith and maerl forms. It is worth noting that there is no clear definition of a rhodolith bed in terms of coverage and proportion of live *versus* dead nodules. Rhodolith beds have been variously defined as areas with a cover of living coralline thalli >10% (Steller *et al*., 2003) or >30% (OSPAR, 2008), within an area of at least 100 m^2^ according to Rinde *et al*. (2022).

## III. VARIATION IN DIVERSITY AND LIFE TRAITS OF RHODOLITHS ACROSS ENVIRONMENTAL GRADIENTS

The potential of rhodoliths to act as foundation species and to enable facilitation is regulated by their size, shape and abundance – attributes that can vary within and among species. The spatial extent and degree of patchiness of rhodolith beds also influence their ability to initiate a facilitation cascade, as well as the number of levels in a cascade, due to different habitat requirements of the associated species (Gribben *et al*., 2019). Thus, understanding the factors shaping variations in the structure of rhodolith beds is crucial for assessing their role as basal species in facilitation cascades.

Variability in rhodolith distribution, shape and morphotype has long been observed (Bosellini & Ginsburg, 1971; Bosence, 1983; Basso, 1998). Rhodolith nodules can be composed of one single morphotype (e.g. maerl beds composed of variably shaped unattached branches; Bosence, 1983; Basso *et al*., 2016), by mixtures of coralline morphotypes (Bracchi *et al*., 2022; Vale *et al*., 2022) or by coralline and *Peyssonnelia* nodules. There are examples of highly diverse rhodolith beds, such as that around Punta de la Mona (western Mediterranean), which is formed by 25 morphospecies belonging to six genera (*Lithophyllum*, *Spongites*, *Neogoniolithon*, *Lithothamnion*, *Mesophyllum* and *Phymatolithon*) (Del Rio *et al*., 2022).

The heterogeneity of the physical structure of rhodolith beds can be described with a ternary diagram that considers the three main morphotypes of nodules [pralines, unattached branches or boxwork (Basso, 1998; Basso, Nalin & Nelson, 2009)], allowing a visual description of the rhodolith bed (Basso *et al*., 2016; Bracchi *et al*., 2022; Caronni *et al*., 2023). The shape of rhodoliths is often measured following the criteria of Sneed & Folk (1958), which requires measuring the longest, intermediate, and shortest (axial) diameters (Sciberras *et al*., 2009) and using these measures to classify nodules into four classes – spheroidal, discoidal, ellipsoidal or bladed (Bosence, 1976; Gagnon, Matheson & Stapleton, 2012; Carro *et al*., 2014; Villas‐Bôas *et al*., 2014; Otero‐Ferrer *et al*., 2020; Neves *et al*., 2021) – as well as to calculate sphericity (Voerman *et al*., 2022).

Light, temperature, hydrodynamics and sedimentation are among the main factors regulating the characteristics of rhodolith assemblages (Basso, 1998; Carvalho *et al*., 2020; Otero‐Ferrer *et al*., 2020; Sissini *et al*., 2022). Rhodolith size and shape are mostly regulated by hydrodynamics, whilst light limitation or burial under sediments can stunt growth (Villas‐Bôas *et al*., 2014; Bracchi *et al*. 2019; Omachi *et al*., 2019). Features of coastlines (e.g. presence of sheltered bays or inlets) and the extension of the continental shelf influence these physical factors and, hence, may play a role in regulating the size, shape and density of rhodolith nodules. For example, along bathymetric gradients, the largest rhodoliths have been found in the shallowest areas at some sites (Bahia *et al*., 2010; Voerman *et al*., 2022), while they are located more often in intermediate or deepest areas sampled at other sites (Sañe *et al*., 2016; Del Rio *et al*., 2022; Perez‐Peris *et al*., 2023). This suggests that location‐specific environmental conditions can be more important than factors covarying with depth (e.g. light, temperature, sedimentation and hydrodynamic forces) in determining rhodolith size and/or abundance. However, in the northeast Atlantic, the shape of rhodoliths and complexity of the bed they formed were weakly influenced by bottom currents and wind exposure, and driven instead by underlying sediment composition (Jardim *et al*., 2022). In addition, the shape of rhodoliths is influenced by biotic factors. For example, boring bivalves can produce cavities in nodules, providing further niche space for both vertebrates and invertebrates (Gagnon *et al*., 2012; Teichert, 2014).

## IV. POTENTIAL OF RHODOLITHS TO MODIFY BIOTIC AND ABIOTIC CONDITIONS

Rhodoliths are widely recognised as ecosystem engineers (Nelson, 2009; Teichert & Freiwald, 2014; Qui‐Minet *et al*., 2018; Otero‐Ferrer *et al*., 2019; Voerman *et al*., 2022) and there is compelling evidence that they support higher invertebrate species diversity compared to surrounding soft sediments (Steller *et al*., 2003; Teichert, 2014; Boye *et al*., 2019; Neves & Costa, 2022) and equivalent fish species diversity compared to adjacent coral reefs (Moura *et al*., 2021; Anderson *et al*., 2023). Nonetheless, few studies have formally identified the mechanisms underpinning facilitation, which requires experimental manipulation of biotic (e.g. manipulation of consumer or competition pressure) and/or abiotic conditions (e.g. environmental stressors) (Bulleri, 2009; Thomsen *et al*., 2018, 2022; Gribben *et al*., 2019). Instead, the positive effects of rhodoliths on other species have been widely explained through the general effect of (micro)habitat creation, since nodule and bed complexity generate multiple niches for associated organisms. Below, we provide some examples of rhodolith beds enhancing the settlement, growth and survival of benthic species for each of the three facilitation mechanisms. Note that this is not meant to be a comprehensive review of the literature and here we distinguish among the different facilitation mechanisms for clarity, although they are likely to co‐occur due to simultaneous changes in both biotic and abiotic conditions induced by rhodoliths.

### (1) Amelioration of environmental conditions

Although the amelioration of environmental conditions is not a predominant mechanism of species facilitation in subtidal environments (Bulleri, 2009), intense hydrodynamic forces and high sediment load can generate adverse conditions for benthic species (Witman, 1987; Bulleri *et al*., 2011). Rhodolith beds can thrive in wave‐ and/or current‐swept areas, yet their rigid calcareous thalli can reduce hydrodynamic forces and trap finer sediments, providing shelter and trophic resources to other species (Hall‐Spencer, 1998; Gabara, 2020). Experiments showed that irregularly shaped rhodoliths can enhance substratum stability in wave‐swept environments (Joshi, Duffy & Brown, 2017), regulate sediment grain size distribution (de Queiroz *et al*., 2016) and create interstices that favour oxygen penetration to deeper layers (Hall‐Spencer & Atkinson, 1999), ultimately favouring colonisation by both epibenthic and infauna species (Caronni *et al*., 2023). Rhodoliths have also been documented on intertidal flats, but reported densities are too low (<1 m^2^) for the formation of habitat (Perry, 2005), and, hence, their potential to facilitate species through the buffering of heat and desiccation stress remains uncertain.

Coralline algae, including rhodoliths, alter seawater chemistry through their metabolic activity (i.e. photosynthesis, respiration and calcification) by producing O_2_, and modifying pH, HCO_3_^−^ and CO_3_^2−^ concentrations at their surface with respect to the surrounding water (Hofmann, Schoenrock & de Beer, 2018; McNicholl, Koch & Hofmann, 2019; Schubert *et al*., 2021). It is likely that these changes in seawater chemistry provide more suitable conditions for other non‐calcifying species, at least during daytime. This feature is likely to become even more relevant under projected levels of ocean acidification and warming (Cornwall *et al*., 2022).

### (2) Reduction of predation and competition pressure

By providing a three‐dimensional structure, rhodolith beds act as nursery areas for both vertebrate and invertebrate species, including those of commercial importance; this is likely due to the reduction of competition pressure they provide *via* refuge creation (Kamenos, Moore & Hall‐Spencer, 2004a). For example, juvenile cod (*Gadus morhua*), saithe (*Pollachius virens*) and pollack (*Pollachius pollachius*) were more abundant in rhodolith habitats than adjacent heavily vegetated rocky and gravel substrata (Kamenos, Moore & Hall‐Spencer, 2004c). Rhodolith beds have a high holding capacity for juvenile gadoids, likely by virtue of their food and refuge provisioning, and are thus an important part of the inshore nursery system (Kamenos, Moore & Hall‐Spencer, 2004d). The sand tilefish *Malacanthus plumieri* is almost exclusively found on rhodolith beds, building rhodolith mounds that are used as a reference during foraging movements, shelter against predation and may also play a role in social organisation (Pereira *et al*., 2015). The mounds themselves are used as microhabitats for several other fish and invertebrate species (Pereira *et al*., 2015; Francini *et al*., 2018). Similarly, hollow rhodoliths, formed by boring bivalves, are known to function as nesting sites for fish and for shelter from predation for several species of ophiurids (Gagnon *et al*., 2012; Teichert, 2014). The positive effects of rhodoliths through the reduction of consumer pressure are not limited to the formation of interstices that can be used by prey as refuges from predators. Leemans *et al*. (2020) demonstrated experimentally that spiky rhodoliths promoted the recovery and persistence of the seagrass *Thalassia testudinum* by directly reducing the access of marine turtles to plants. Likewise, juvenile bivalves of many species have been found at higher densities in rhodoliths compared to surrounding substrata (Kamenos *et al*., 2004c; Steller & Caceres‐Martinez, 2009). This is thought to be due to attraction to the living coralline algal surface and also the presence of a rugose three‐dimensional structure (Kamenos, Moore & Hall‐Spencer, 2004b).

There is evidence that the presence of the living algal veneer acts as an attractant to juvenile scallops, signalling refuge presence and endowing lower stress responses in the presence of predatory starfish (Kamenos, Calosi & Moore, 2006). The attractant effect may be facilitated by the high production of dimethylsulphide (DMS) and its secondary metabolite precursor dimethylsulphoniopropionate (DMSP) by rhodoliths, especially at high and low latitudes (Burdett, Hatton & Kamenos, 2015; Burdett, 2017). Both compounds are important chemical cues for a range of ecological processes, including herbivorous grazing and vertebrate larval settlement (Lyons, Scheibling & Van Alstyne, 2010; Foretich *et al*., 2017).

### (3) Enhancement of resource availability

Biogenic substrata, primarily formed by rhodoliths and often consolidated by binding species, such as sponges or mussels, may extend the distribution of hard‐bottom‐dwelling species to soft sediments, which can play an important ecological role in places with reduced availability of natural rocky bottoms (Ávila, Riosmena‐Rodriguez & Hinojosa‐Arango, 2013; Pereira *et al*., 2015). This phenomenon has been recorded in estuaries, deep reefs in oceanic islands with reduced shelf and on seamounts (Steller *et al*., 2003; Pereira *et al*., 2012; Otero‐Ferrer *et al*., 2019). For example, in the Madeira Archipelago, rhodolith beds considerably increase the availability of subtidal hard substrata around the islands (Neves *et al*., 2021). The provisioning of consolidated, biogenic substrata could be highly relevant also on hard bottoms since, despite the availability of hard surfaces, the settlement and growth of benthic species can be facilitated or, indeed, restricted to living coralline algal beds (Kamenos *et al*., 2004a; Steller & Caceres‐Martinez, 2009). Vertical expansions of species distribution (i.e. bathymetric) fostered by rhodoliths might be particularly relevant in the context of global warming (see Section VI).

Rhodolith beds increase food availability for associated organisms by entraining organic matter and promoting local small‐scale primary production from microphytobenthos and soft red seaweeds, thus increasing secondary production (Grall *et al*., 2006; Gabara, 2020; Neto, Bernardino & Netto, 2021; Teper, Parrish & Gagnon, 2022). This is consistent with analyses of the trophic groups associated with rhodolith beds, revealing a dominance of deposit feeders (Grall *et al*., 2006; Sciberras *et al*., 2009; Teper *et al*., 2022). The higher resource availability and diversity of food sources in rhodolith beds may ensure a more constant resource supply than in bare habitats. Indeed, stable isotope analyses have suggested that Arctic rhodolith beds may function as benthic–pelagic hotspots, at least seasonally (Teper *et al*., 2022). Finally, the high functional richness and redundancy of associated communities (Boye *et al*., 2019) may promote temporal community stability through compensatory dynamics and asynchronous species temporal fluctuations within functional groups (Magurran & Henderson, 2018).

## V. MULTIPLE FACILITATION CASCADES IN RHODOLITH BEDS

Many of the species or groups of species that are dependent upon rhodoliths for colonisation of soft sediment areas can act as secondary facilitators. According to the Foundation Species‐Biodiversity (FSB) model (Angelini & Silliman, 2014), the difference in morphological traits between the basal and the secondary facilitator determines the strength of the effects on species diversity. The larger the difference, the greater the positive effect of the association between the basal species and secondary facilitators on species diversity at upper cascade levels. By contrast, the addition of a secondary facilitator that does not differ from the basal species in terms of morphological complexity would simply increase habitat availability, enhancing the abundance, but not the diversity, of species within the assemblage associated with the basal species. The effects of secondary facilitators on species abundance and diversity can be expected to be very high in rhodolith beds since they support a broad variety of secondary facilitators. Rhodolith beds are often composed of different rhodolith morphospecies (up to 25 in a single bed; Del Rio *et al*., 2022), with subsequent large variations in functional traits (e.g. size, shape, rugosity, branching) and thereby, high microhabitat diversity. Indeed, although unexplored, some rhodolith morphospecies could facilitate others. For example, highly branched forms could enhance the retainment of simpler nodules, by reducing their drag by currents. This means that, conceptually, the first two levels of a facilitation cascade (i.e. the basal species and the secondary facilitator) could both be represented by rhodoliths. High within‐bed rhodolith diversity should therefore broaden the pool of species they can support at the seascape scale and, hence, increase the likelihood of secondary facilitator establishment. In addition, a single rhodolith bed can support multiple taxonomically distant and architecturally distinct species (e.g. Porifera, Cnidaria, Mollusca, Ochrophyta, Chlorophyta, other Rhodophyta), with a broad range of functional traits and hence, a wealth of secondary microhabitat and food resources provision. Rhodoliths, by supporting many secondary facilitators that differ morphologically, are expected to amplify positive effects on species diversity.

Two features that distinguish rhodoliths from most foundation species are their small size and mobility. Many of the widely acknowledged marine foundation species, including seagrasses, mangroves, kelps and corals, are generally conspicuous in size and larger than the species they support through their facilitative effects. Thus, in most cases, there is a clear decreasing pattern in body size when moving from the basal species to the upper levels of facilitation cascades. This does not necessarily apply to rhodoliths, as they are often smaller than the species they support that act as secondary facilitators, such as many seaweeds, sponges and bivalves. Thus, despite being dominant in abundance, and at the base of a network of positive, non‐trophic species interactions (in accordance with the definition by Ellison, 2019), rhodoliths markedly deviate from the common view of foundation species. Although unexplored, the smaller size of rhodoliths – in comparison to that of the species they support – could increase the likelihood of negative feedbacks. For example, epibiota (e.g. sponges, oysters) and epiphytes (macroalgae) on the roots of mangroves (Bishop *et al*., 2012; Gribben *et al*., 2019) are unlikely to have negative effects on the trees, while they could completely overgrow rhodoliths, hampering their movement and impairing their photosynthetic efficiency.

Rhodoliths can be transported by waves and currents (Lavenère‐Wanderley *et al*., 2021). The only other known example of a mobile foundation species is that of the pencil sea urchins in the Galapagos (Altieri & Witman, 2014). The mobility of rhodoliths implies that they can redistribute associated species, potentially expanding the distribution of species with short‐distance dispersal abilities. Widespread rolling and movement of rhodoliths occurs sporadically because of storms, but also periodically, due to currents and wind‐propagated waves [up to several centimetres per day (Steller & Foster, 1995; Harris *et al*., 1996; Marrack, 1999)]. Overgrowth by large erect species, such as macroalgae and sea fans, is likely to increase the distance over which rhodoliths can be dragged by currents and waves. Moreover, associated fauna can contribute to rhodolith displacement through bioturbation, for example movement of sea urchins that use rhodoliths as covering material (Foster *et al*., 1997; Marrack, 1999) and the activity of the sand tilefish that moves rhodoliths to build mounds, likely playing an important role in the spatial extension of rhodolith beds (Pereira *et al*., 2015). Displacement by biotic or abiotic factors does not transport rhodoliths exclusively horizontally, but can cause their spilling down from shallow‐water environments to greater depths, such as in the case of steep slopes of seamounts, where rhodoliths have been found at a depth of up to 290 m (Littler, Littler & Hanisak, 1991). On the other hand, some epifauna (e.g. sponges, tunicates, anemones) can also bind rhodoliths together, decreasing their movement (Marrack, 1999).

Although rhodolith survival is dependent upon light availability, their calcified skeleton means that even dead nodules maintain their shape and can continue to support a high diversity of associated species (Kamenos *et al*., 2004, 2004). As acknowledged for other foundation species (Saldaña *et al*., 2024), rhodoliths are therefore likely to maintain their foundation species effect beyond their lifetime and in areas unsuitable for their survival.

**Fig. 2:**
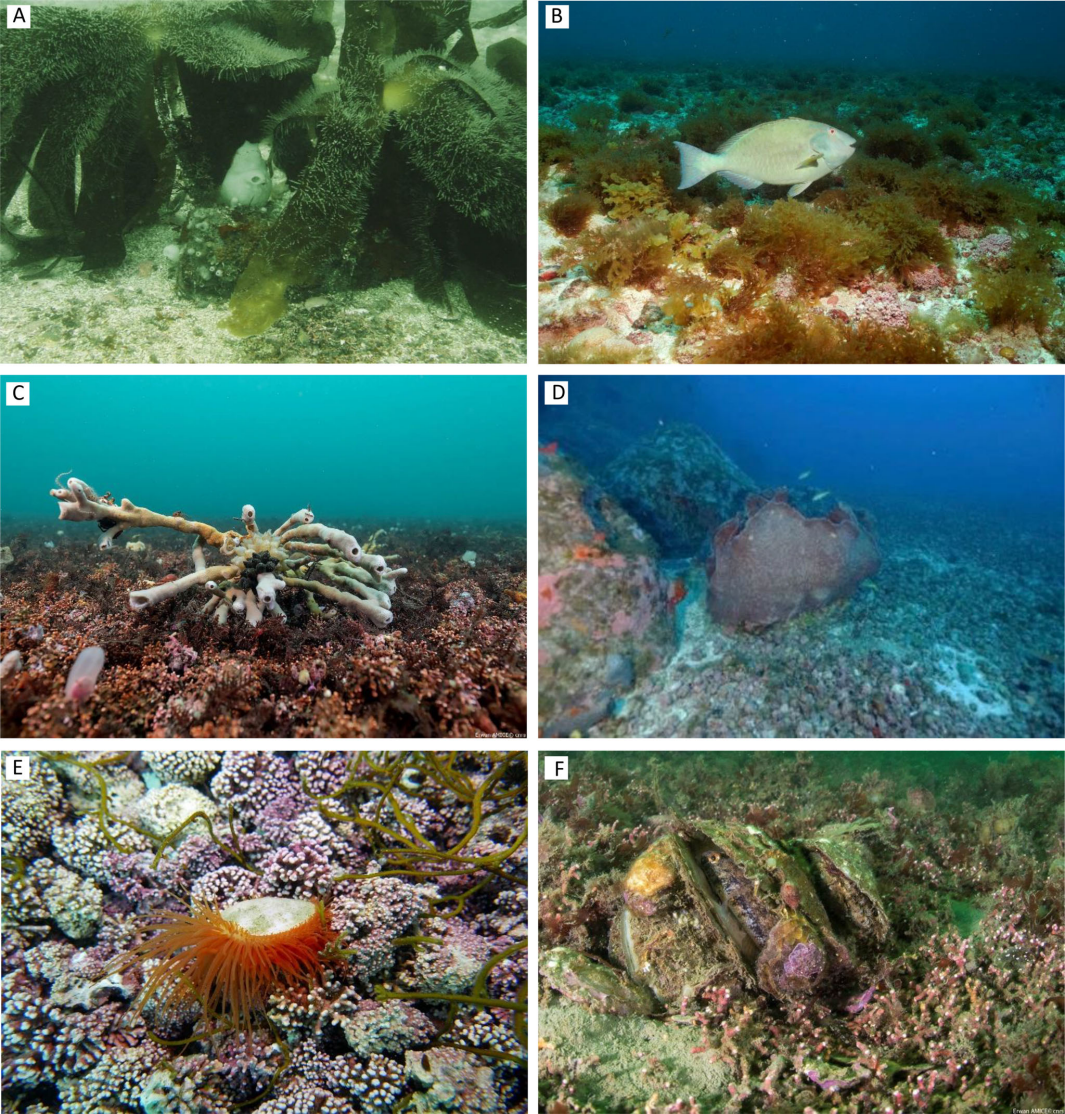
Habitat‐forming species in rhodolith/maerl beds. (A) *Laminaria ochroleuca* from Galicia, Spain, depth 11 m (photograph credit: Ignacio Bárbara. (B) Diverse macroalgal assemblages supported by rhodoliths at the Fernando de Noronha Archipelago, Brazil, depth 40 m (photograph credit: Zaira Matheus). (C) The tubular sponge, *Haliclona simulans*, supporting ophiurans, gastropods and hosting cuttlefish eggs in Brittany, France, depth 7 m (photograph credit: Erwan Amice). (D) Unidentified concave sponge from the Fernando de Noronha Archipelago, Brazil, depth 40 m (photograph credit: Zaira Matheus). (E) The bivalve *Limaria hians* in the north of Norway, depth 15 m (photograph credit: Jason Hall‐Spencer). (F) The flat oyster *Ostrea edulis* supporting fish spawning, hydroids, seaweeds, encrusting sponges, ascidians and galatheidaes in a maerl bed of Brittany, France, depth 3 m (photograph credit: Erwan Amice).

To the best of our knowledge, no study has, to date, experimentally demonstrated facilitation cascades (i.e. the co‐occurrence of the basal species, i.e. the primary facilitator, and a secondary facilitator or a focal species that does not form habitat) in rhodolith beds. Nonetheless, based on the available information and to illustrate the potential of rhodoliths to start facilitation cascades, we here focus on three taxa, seaweeds, sponges and bivalves (Fig. 2), that are commonly associated with rhodoliths and are broadly known to function as primary or secondary facilitators (Dayton, 1972; Steneck *et al*., 2002; Gribben *et al*., 2009; Bishop *et al*., 2012; MacDonald & Weis, 2013; van de Koppel *et al*., 2015; Thomsen *et al*., 2018, 2022; Ravaglioli *et al*., 2021). This does not exclude the potential for other taxa to be part of rhodolith facilitation cascades. Moreover, our examples are focused on facilitation cascades in which the rhodoliths and the secondary benefactors are embedded within the same patch (i.e. nested facilitation, *sensu* Angelini *et al*., 2011). However, as shown for other facilitative interactions, such as those involving mangroves, seagrasses, corals, oysters and marsh plants (van de Koppel *et al*., 2015; Gribben *et al*., 2019), positive effects of rhodoliths could expand beyond the margins of the bed they form to influence other habitats (i.e. adjacent or landscape facilitation, *sensu* Angelini *et al*., 2011). For instance, some of the biomass generated by invertebrates and macroalgae preferentially recruiting into rhodolith beds might move outside, either actively or passively, fuelling adjacent bare‐sand trophic webs. Likewise, rhodolith‐driven changes in water chemistry (i.e. pH and CO_3_^2−^) or bottom currents could affect adjacent habitats, such as coralligenous or coral reefs and seagrass beds.

### (1) Seaweeds

Seaweed assemblages associated with rhodolith beds are known to be extremely diverse. For instance, northeast Atlantic maerl beds host at least 349 associated macroalgal species, including 232 Rhodophyta, 72 Heterokontophyta and 45 Chlorophyta, making up about 30% of the total macroalgal diversity in the region (Peña *et al*., 2014). Likewise, Helias & Burel (2023) recorded 170 macroalgal species, belonging to Rhodophyta, Ochrophyta and Chlorophyta in rhodolith beds from the Bay of Brest, 108 of which were found growing exclusively on rhodoliths, while 14 occurred only as epiphytes on other species, indicating two and three levels of co‐occurring habitat‐formers. In Brazil, local estimates (across areas of tens of km^2^) of seaweed diversity in rhodolith beds range from 44 to 67 species, with regional estimates (i.e. across hundreds of km^2^ in the Abrolhos Bank) of 146 species (Riul *et al*., 2009; Pascelli *et al*., 2013; Brasileiro *et al*., 2016).

**Fig. 3:**
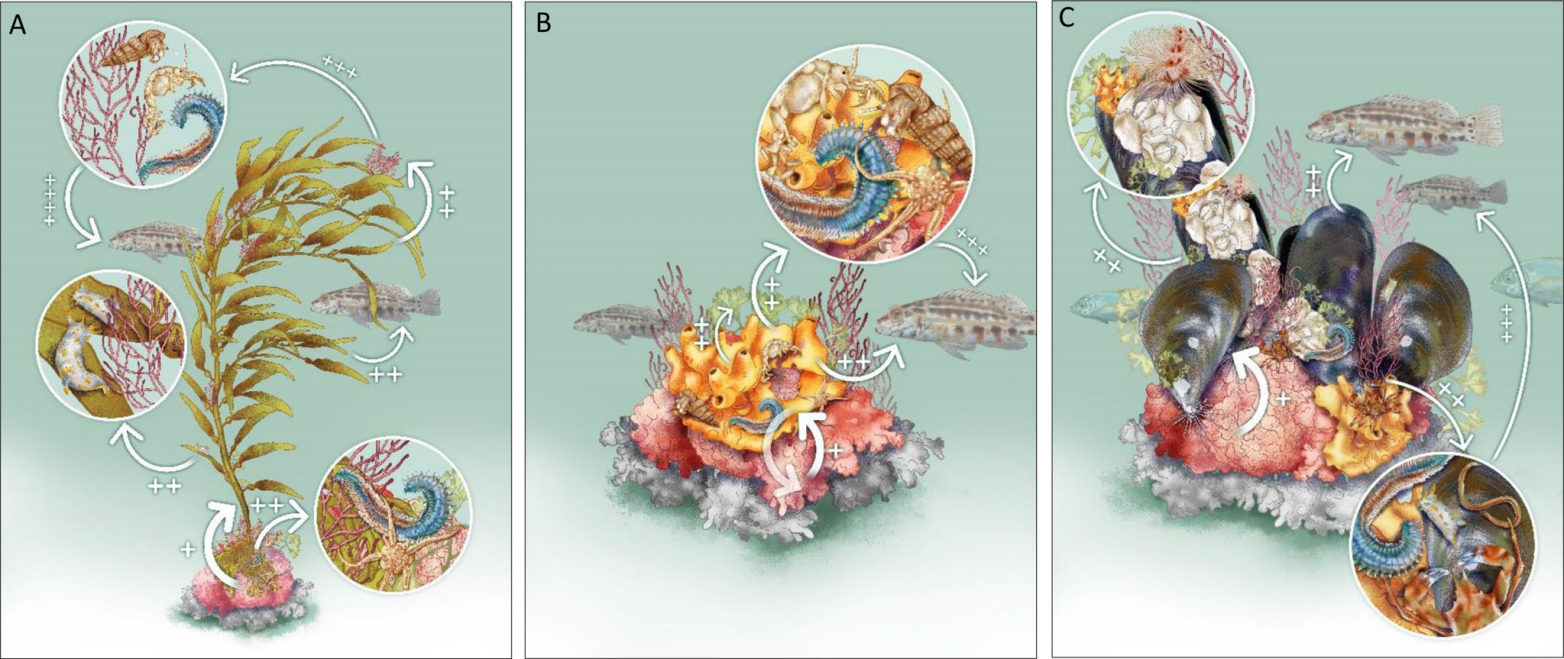
Positive interactions within facilitation cascades in rhodolith beds. (A) Rhodoliths (the basal species) promote the establishment of kelp on sedimentary bottoms through the provisioning of hard substrates for attachment. The holdfast, stipe and blades of kelp (secondary facilitator) can provide habitat and/or food to fish and invertebrates, directly or indirectly by supporting epibiota (e.g. macroalgae) that act as tertiary facilitators. (B) Rhodoliths facilitate the establishment of sponges which, in turn, can stabilise beds through the binding of nodules. Sponges (secondary facilitators) can provide habitat for a variety of invertebrates and sustain the growth of macroalgae by recycling nutrients. (C) Bivalves growing on rhodoliths can facilitate invertebrates and macroalgae by providing interstitial or attachment space. In all cases, species at the second or upper level within the facilitation cascade can be used as food by consumers (i.e. herbivores or predators). Arrows show the positive effects of one species on another, with the number of plus signs indicating the level of the facilitative interaction within the cascade. Some of the functions performed by rhodoliths, such as the creation of microhabitat and provisioning of attachment surface, can continue when they are dead (represented in grey); for convenience, dead rhodoliths are illustrated below the living layer, although, in real beds, the surficial layer is often composed of a variable proportion of live and dead nodules. Magnifying lenses provide details of macroalgae and invertebrates supported by secondary facilitators (Illustration by ©Lúcia Antunes, www.luciaantunes.com).

Rhodolith beds also host kelp species (Fig. 2A), which are themselves foundation species (Steneck *et al*., 2002; Fernandez, 2011; Bracken, 2018). Association of kelp species (e.g. *Saccharina latissima*, *Saccorhiza polyschides*, *Laminaria hyperborea*, *L. abyssalis*, *L. ochroleuca*, *L. rodriguezii*, *Ecklonia radiata*) with rhodoliths has been documented in both tropical and temperate basins, including the Mediterranean, and the coasts of France, New Zealand, Brazil and the Madeira islands (Amado *et al*., 2007; Peña & Bárbara, 2008; Nelson, 2009; Amado‐Filho & Pereira‐Filho, 2012; Barberá *et al*., 2012; Marins *et al*., 2014; Peña *et al*., 2014; Braga‐Henriques *et al*., 2022). In some cases, such as that of *L. rodriguezii* in the Menorca Channel (western Mediterranean), kelps can comprise most of the macroalgal biomass associated with rhodolith beds. While large kelp individuals, and the rhodoliths to which their haptera are attached, can be dislodged from rhodolith beds in shallow, wave‐swept areas, kelp populations on deeper beds are likely to be more stable. For example, deep (45–120 m) rhodolith beds of the Abrolhos Bank (Brazil) host permanent populations of *L. abyssalis* (Amado‐Filho & Pereira‐Filho, 2012; Foster *et al*., 2013). Thus, rhodolith beds can extend the distribution of this marine foundation species to areas that would otherwise lack suitable hard surfaces (Foster *et al*., 2013), creating a facilitation cascade that enhances local and regional species diversity (Fig. 3A).

Epiphytic macroalgae supported by rhodoliths, including filamentous and fleshy morphotypes (Fig. 2B), can also provide shelter against predators and resources (space and food) for fish (Chaves, Pereira & Feitosa, 2013; Fulton *et al*., 2020) and for sessile and mobile invertebrates, as widely demonstrated for epiphytes on seagrasses and macroalgae (Thomsen *et al*., 2018; Ravaglioli *et al*., 2021; El‐Khaled *et al*., 2022). Thus, although not considered as foundation or habitat‐forming species, epiphytic macroalgae can still function as secondary or tertiary facilitators (Fig. 3). In addition, some macroalgal species, such as *Codium bursa* and *C*. cf. *effusum*, were exclusively found free‐living (i.e. unattached to the bottom) within rhodolith beds in the Bay of Brest, France (Helias & Buriel, 2023) and in Sardinia (D. Basso, personal observations). This suggests that facilitation can occur at scales varying from that of the single rhodolith (by providing a surface for attachment) to that of the whole bed, which, by virtue of its complex topography, acts as a passive collector of macroalgae, comparable to mangrove pneumatophores in tidal flats (Bishop *et al*., 2012; Bastos *et al*., 2013). This may be important in sustaining a large biomass of fleshy organic carbon which can then be buried locally in the rhodolith bed, thus acting as a blue carbon repository (James *et al*., 2024; Mao *et al*., 2020). Finally, rhodolith beds may also function as a bank for microscopic algal stages, thus representing a reservoir for several macroalgae (Hoffmann & Santelices, 1991; Fredericq *et al*., 2019).

### (2) Sponges

As described above for seaweeds, rhodolith beds host highly diverse sponge assemblages (Fig. 2C, D), including boring (infauna) and epifaunal species, across the world's oceans (Solórzano & Urgorri, 1991; Aguilar *et al*., 2009; Ávila *et al*., 2013; Pereira‐Filho *et al*., 2015; Longo *et al*., 2020). For example, in two rhodolith beds off the Island of Ustica (Southern Tyrrhenian Sea), Longo *et al*. (2020) documented the presence of 25 sponge taxa. While mostly belonging to the class of Demospongiae, these sponges were characterised by a variety of growth forms (i.e. massive, encrusting, insinuating and excavating). Similarly, Solórzano & Urgorri (1991) reported 39 species of sponges associated with a single Galician maerl bed, while Santín *et al*. (2024) documented eight sponge species in rhodolith beds within the Madeira archipelago, including a new species (*Hemimycale funchalensis*). Recent surveys in mesophotic rhodolith beds off the Amazon River mouth in Brazil have recorded highly diverse sponge assemblages, including new species (Moura *et al*., 2016; Sandes *et al*., 2021). Such “sponge gardens” over rhodolith beds in north Brazil, in turn, serve as habitat for a diverse fish assemblage and may act as stepping stones for reef biota between the Caribbean and Brazilian Provinces (Rocha, Rosa & Feitoza, 2000; Rocha, 2003).

When at high density and biomass (Lopez‐Acosta *et al*., 2022), sponges can form structurally complex structures that provide nursery and rearing areas for other organisms, often augmenting the diversity of invertebrate and fish assemblages (Fig. 3B) (Rocha *et al*., 2000; Kazanidis *et al*., 2016; Hawkes *et al*., 2019; Campanino *et al*., 2023). Sponges are known to be secondary habitat‐formers in mangrove forest facilitation cascades (Gribben *et al*., 2019). For example, the species richness and abundance of fish and mobile invertebrate assemblages in Caribbean mangrove forests was positively correlated with the abundance of sponges growing on prop‐roots (MacDonald & Weis, 2013; Stewart *et al*., 2022). Sponges with a tubular, convoluted or massive growing form could provide shelter from predation for smaller fish and, at the same time, represent a food source for other fish or invertebrate species. In oligotrophic systems, sponges can also exert positive effects on primary producers through nutrient supply (Archer *et al*., 2021) – providing a growth‐promoting feedback loop to the rhodoliths and other associated algae.

In addition, sponge–rhodolith assemblages play an important role in substrate construction and stabilisation, due to overgrowth and binding (Ávila *et al*., 2013; Pereira‐Filho *et al*., 2015). For example, on the continental shelf of the Fernando de Noronha Archipelago, rhodolith mounds formed by the sand tilefish can then be bound by the sponges *Xestospongia muta* and *Agelas clathrodes*, stabilising the rhodolith accumulation and facilitating the establishment of coral colonies (Pereira *et al*., 2015; Pereira‐Filho *et al*., 2015). Within this context, rhodoliths directly enable at least two distinct cascades – one initiated by the sand tilefish and the other by sponges, with cascading effects across trophic levels.

### (3) Bivalves

Bivalves are common inhabitants of rhodolith beds (Fig. 2E, F), both as epifauna and endofauna (Kamenos *et al*., 2004c; Hall‐Spencer *et al*., 2003; Steller *et al*., 2003) and can also act as secondary facilitators (Fig. 3C). Several aggregating bivalve species, such as flat oysters or mytilid species, may reach densities high enough to enhance further the three‐dimensional complexity of rhodolith beds (Fig. 3C), promoting local biodiversity through the provisioning of refugia against predators or by trapping organic matter (Norling & Kautsky, 2008; Bishop *et al*., 2012; Gribben *et al*., 2019), as also shown for oysters growing on mangrove prop‐roots (Bishop *et al*., 2012; Stewart *et al*., 2022). Rhodolith beds are able to support bivalve species in areas where they are otherwise locally rare, such as enhanced abundance of *Gregariella semigranata* and *Leisonelus aristatu* in the oligotrophic waters of the Madeira Archipelago (AMACO, 2022). Similarly, in the northeast Atlantic, *Limaria hians*, a bivalve species of high conservation value, uses rhodolith beds for nesting, again stabilising the substratum and facilitating further colonisation by sessile species (Hall‐Spencer & Moore, 2000). Further species diversity is facilitated in rhodolith beds *via* support of larger bivalve species such as fan mussels, horse mussels and scallops (Steller & Caceres‐Martinez, 2009; Kersting & García‐March, 2017), which host additional distinctive species assemblages on their shells, including macroalgae, hydroids, sponges, molluscs, bryozoans, and crustaceans (Corriero & Pronzato, 1987; Cummings *et al*., 1998; Giacobbe, 2002; Cerrano *et al*., 2001; Farren & Donovan, 2007) (Fig. 3C).

## VI. RESEARCH NEEDS AND FUTURE DIRECTIONS IN THE CONTEXT OF FACILITATION CASCADES

Below, we provide a synthetic account of strategic research required to fill in gaps in our understanding of the role of positive species interactions in shaping the biodiversity and functioning of rhodolith beds.

### (1) Experimental evaluation of the role of rhodoliths as basal facilitators

**Fig. 4:**
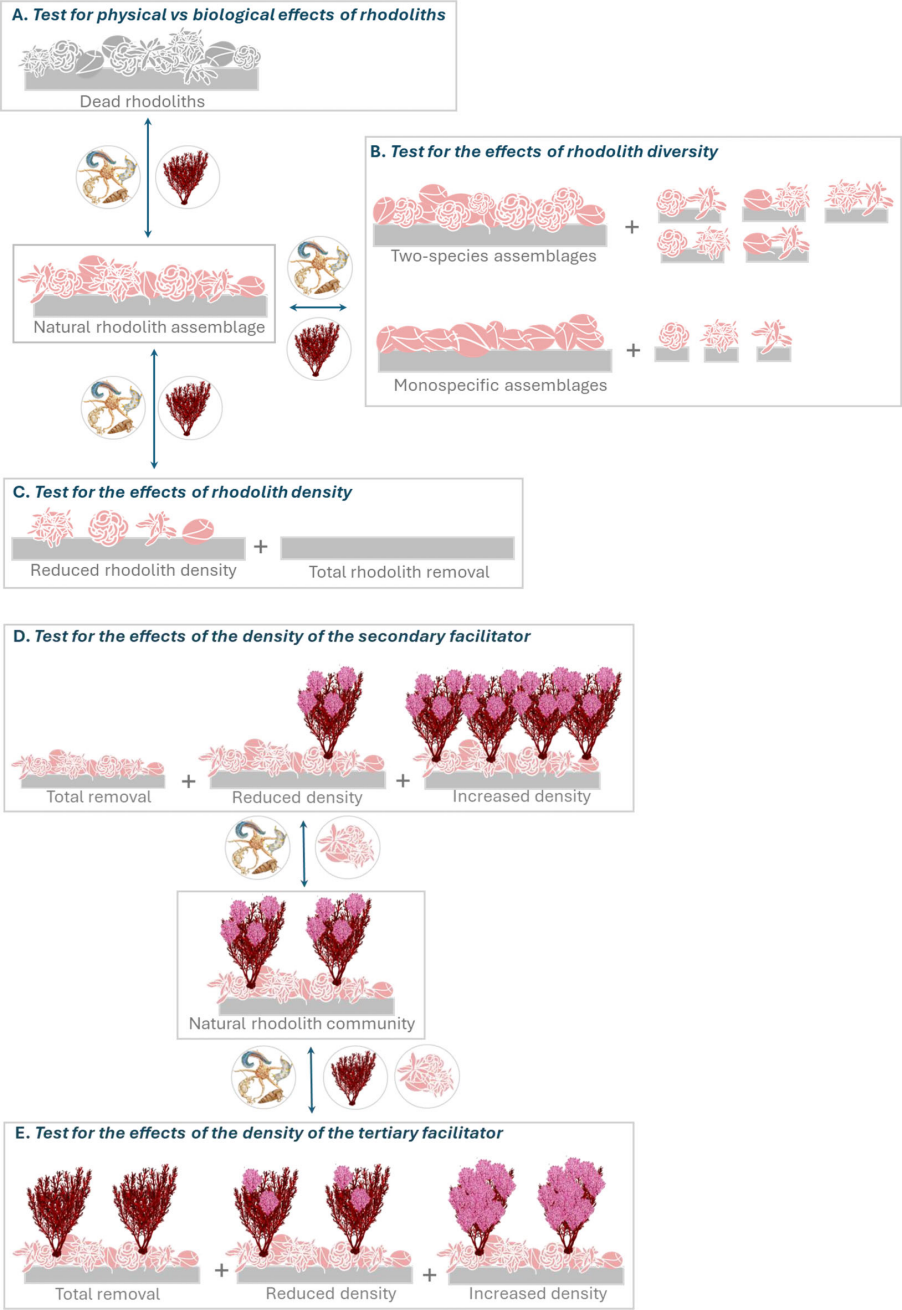
(A–C) Schematic representation of potential experiments assessing the effects of rhodolith bed traits on associated species. (A) A comparison between live and dead rhodoliths. (B) Comparisons among rhodolith assemblages composed of a different number of species or morphospecies (e.g. natural assemblage *versus* two‐species assemblages *versus* monospecific assemblages); in this example, natural rhodolith assemblages are composed of four species across a branching gradient. (C) Comparisons between assemblages composed of the same rhodolith species, but differing in their density (natural *versus* reduced *versus* total removal). (D, E) Schematic representation of potential experiments assessing the effects of upper‐level facilitators on rhodoliths and associated assemblages. (D) Different densities of the secondary facilitator (natural *versus* total removal *versus* reduced density *versus* increased density). (E) Different densities of the tertiary facilitator (natural *versus* total removal *versus* reduced density *versus* increased density); in these examples, the secondary and tertiary facilitators are a canopy‐forming and an epiphytic macroalga, respectively. For each of the illustrated experimental tests, double‐headed arrows indicate comparisons with the natural controls and lateral close‐ups represent the response variable/s (the rhodoliths, the canopy‐forming macroalga and the invertebrate assemblage) that can be potentially taken into account to assess positive/negative effects of each of the three levels included in the cascade. The illustrated densities of the basal, secondary and tertiary facilitators are simple examples of possible manipulations which can be extended to the upper levels of a cascade. A detailed account of experimental designs for disentangling the effects of species richness from those of species identity and density can be found in Benedetti‐Cecchi (2004).

Due to the hierarchical nature of the organisation of rhodolith communities, assessing the effect of the foundation species (i.e. the rhodoliths) on species at upper levels of the facilitation cascade should be considered a priority. To the best of our knowledge, no study has assessed whether the facilitative effects of rhodoliths are biological and/or physical (Fig. 4A). Only one study (Otero‐Ferrer *et al*., 2019) has experimentally investigated the effects of variations in the size and heterogeneity of nodules on macrofaunal assemblages and the role of rhodolith species composition in regulating the structure of associated invertebrate and macroalgal communities (Fig. 4B) remains virtually unexplored. The removal of rhodolith nodules from some areas, or transplanting them onto bare sediments, to generate different densities (Fig. 4C), would allow assessments of their net effect on the diversity of the associated community and to identify which species are reliant on their presence (i.e. obligate associations). In addition, the experimental manipulation of rhodolith species richness and abundance could provide insights into the ecological mechanisms sustaining the functioning of the associated community (Tilman, 1999; Lehman & Tilman, 2000; Loreau, 2000). According to the Biodiversity–Ecosystem Function theory (Hooper *et al*., 2012; Naeem, Duffy & Zavaleta, 2012), the number and relative abundance of facilitated species would be expected to increase with the number of rhodolith species or morphologies composing a bed. Due to their mobile nature and reduced size, the experimental manipulation of rhodoliths is more feasible than those of other coastal foundation species that are generally large sized, sessile and, in some cases, have developed root systems (e.g. seagrass and mangroves). Rhodolith manipulation, among areas within beds or between beds and nearby bare habitats, over relatively small areas would be sufficient to assess the effects on associated flora and fauna without causing significant damage to beds. On the other hand, experimental manipulations by scuba divers could be limited by depth in some regions. For example, in the Mediterranean Sea, many rhodolith beds occur at depths greater than 50 m (Basso *et al*., 2017; Illa‐López *et al*., 2023). However, rapid technological advancements in underwater remote operating vehicles may provide novel opportunities for experimental research on deeper habitats in the near future.

### (2) The length of facilitation cascades and negative feedbacks on rhodoliths

We currently have no empirical data on the number of levels within rhodolith bed facilitation cascades. Experimental removal approaches will be needed to assess the role of secondary facilitators on upper levels of the facilitation cascade (Thomsen *et al*., 2016). The analysis of features of secondary and upper‐level facilitators, such as their abundance (Fig. 4D, E) and the degree of morphological complexity, could enhance our understanding of their roles within the cascade (Thomsen *et al*., 2022). In particular, assessing whether there are density thresholds for the formation and maintenance of facilitation cascades should be a priority (Fig. 4D, E). Secondary facilitators may be ineffective at sustaining other species when at very low abundance, or may trigger negative feedbacks on the foundation species (i.e. exert negative effects on rhodoliths) when at very high abundance. For example, in deep waters, high covers of facilitated macroalgae will likely have a detrimental effect on rhodolith nodules through shading and competition for light. In shallow waters, high epiphyte loads may favour the persistence of rhodoliths by mitigating excessive light intensity (Ravaglioli *et al*., 2021), but also increase drag forces due to wave action, potentially leading to onshore stranding (J. Grall, personal observations). Also, the permanent stabilisation of rhodolith beds by bivalves or sponges may be detrimental to underlying rhodolith nodules (Ávila *et al*., 2013), with anecdotal concerns that flame shell *Limaria hians* nests are beginning to smother rhodolith habitat in northwest Scotland. Similarly, the invasive gastropod *Crepidula fornicata* has been reported to overgrow living rhodoliths, leading to smothering and ultimately their death (Grall & Hall‐Spencer, 2003). Likewise, boring species of sponges and bivalves, while generating further microhabitats, can cause nodule fragmentation when at high densities. Thus, as shown for other systems, density‐dependent switches from facilitation to parasitism or competitive exclusion can be expected (Bulleri *et al*., 2011; Schob *et al*., 2014), resulting in a low density of living rhodolith thalli and a decline or loss of the rhodolith bed. Nonetheless, potential negative effects of facilitated species on rhodoliths remain unexplored.

### (3) Variability across environmental gradients

The factors that shape rhodolith composition may also directly influence the associated species community and the interactions among species within and across cascade levels. The presence of marked gradients of environmental conditions or resource availability could open avenues for research framed within well‐established theoretical frameworks, such the Stress Gradient Hypothesis (SGH; Bertness & Callaway, 1994). Assessing changes in the sign and strength of interactions among species within a facilitation cascade under varying environmental conditions, such as wave exposure, light intensity, water chemistry, sedimentation rate or consumer pressure, would provide insights into the facilitation pathways underpinning the hierarchical organisation of rhodolith beds. According to the SGH, the intensity of positive effects of rhodoliths on sessile and mobile species through the provisioning of more stable substrates or the trapping of organic matter are predicted to decrease when moving from wave‐swept to sheltered environments. Likewise, the relevance of rhodoliths as refuges against predation would weaken in areas where consumer pressure is low. For example, positive effects of rhodoliths on the seagrass *Thalassia testudinum* should decrease in intensity and eventually shift to negative (i.e. competition), in areas where the abundance of sea turtles and, hence, grazing pressure, is low.

### (4) The temporal stability of cascades

Facilitation cascade stability is predicted to decrease with growing numbers of facilitation links and increase with functional redundancy (Yakovis & Artemieva, 2017; Gribben *et al*., 2019). Due to the high numbers of species supported, functional redundancy within and across facilitation cascade levels could be expected to be high, enhancing its stability. For example, according to the biodiversity–stability theory (Tilman & Downing, 1994), rhodolith beds composed of multiple rhodolith species could absorb external perturbations more efficiently than monospecific beds. Thus, facilitation cascades in multispecies‐rhodolith beds might be more stable than those found in seagrass beds, mangroves and kelp forests, which are often formed by single species. This potential resilience (Allison, 2004) should be addressed experimentally, testing multi‐stressor scenarios encompassing ocean warming, acidification and their interactions with regional and local stressors (Gissi *et al*., 2021). Assessing the temporal stability of rhodolith facilitation cascades requires, however, a mix of experimental and observational research. On the one hand, the selective removal/addition of species within the same facilitation level would allow evaluation of the role of functional redundancy in sustaining stability. This seems particularly relevant in rhodolith beds since they often host multiple species potentially acting as secondary or tertiary facilitators (Fig. 3). On the other hand, temporal series of data encompassing generation turn‐overs of involved species are necessary to calculate key metrics, such as the coefficient of variation and the synchrony of species fluctuations (Lehman & Tilman, 2000; Loreau & de Mazancourt, 2008).

### (5) Rhodolith beds as climate refugia

While there has been considerable interest in assessing the vulnerability of rhodolith beds to climate changes (Noisette *et al*., 2013; Rindi *et al*., 2019; Koerich *et al*., 2021; Costa *et al*., 2023), field and laboratory experiments remain needed to evaluate their role as climatic refugia (Voerman *et al*., 2022). Depth has been hypothesised to provide refugia against warming (Graham *et al*., 2007; Liberman *et al*., 2022) and the provision of consolidated substrata might extend the bathymetric distribution of hard‐bottom dwelling species to depths less influenced by extreme warming events. In addition, rhodoliths support high diversity and biomass of epiphytic macroalgae, which can modify seawater chemistry through their biological activities. Epiphytic macroalgae, due to photosynthesis, could locally increase seawater pH (Burdett *et al*., 2018) and provide spatial and temporal refugia for benthic calcifying species, including rhodoliths facing ocean acidification. In future envisaged high‐*p*CO_2_ conditions, epiphytic macroalgae on rhodoliths are expected to benefit from higher CO_2_ concentrations and primary production to be enhanced in future rhodolith beds (Martin & Hall‐Spencer, 2017). Thus, these habitats could provide suitable environmental (chemical) conditions for their associated calcifying species in future high‐*p*CO_2_ conditions. The wide distribution of rhodolith beds from tropical to polar latitudes (Fragkopoulou *et al*., 2021), is broader than that of many other marine and coastal foundation species. Thus, assessing how the diversity of rhodolith species or morphospecies and that of associated habitat‐formers varies across such broad latitudinal gradients would provide insights into their potential to act as climate refugia.

## VII. CONCLUSIONS

(1) Despite being distributed throughout the photic zone across the global oceans (from tropical to polar regions), and being recognised as biodiversity hotspots, rhodolith beds are yet to be fully appreciated as key marine habitats at the level of seagrass meadows, mangrove and macroalgal forests or coral reefs.
(2) Here, we show that rhodoliths can function as foundation species of multi‐level facilitation cascades, and hence are fundamental for the persistence of hierarchically structured communities within coastal oceans around the world.
(3) Research priorities should now seek to understand better the processes underpinning these community assemblies, the pathways of ecological facilitation and the effects of human and environmental perturbations.
(4) Addressing these priorities will allow the development of evidence‐based policy decisions and elevate rhodolith beds within marine conservation and coastal management strategies.
